# Impact of menopause and diabetes on atherogenic lipid profile: is it worth to analyse lipoprotein subfractions to assess cardiovascular risk in women?

**DOI:** 10.1186/s13098-017-0221-5

**Published:** 2017-04-07

**Authors:** Marília Izar Helfenstein Fonseca, Isis Tande da Silva, Sandra Roberta G. Ferreira

**Affiliations:** 1 Department of Epidemiology, School of Public Health, University of São Paulo, Av. Dr. Arnaldo, 715, São Paulo, SP 01246-904 Brazil; 2 Department of Nutrition, School of Public Health, University of São Paulo, Av. Dr. Arnaldo, 715, São Paulo, SP 01246-904 Brazil

**Keywords:** Menopause, Women, Cardiovascular risk, Lipoprotein subfractions, Diabetes mellitus

## Abstract

Cardiovascular disease is the leading cause of death in women at advanced age, who are affected a decade later compared to men. Cardiovascular risk factors in women are not properly investigated nor treated and events are frequently lethal. Both menopause and type 2 diabetes substantially increase cardiovascular risk in the female sex, promoting modifications on lipid metabolism and circulating lipoproteins. Lipoprotein subfractions suffer a shift after menopause towards a more atherogenic lipid profile, consisted of hypertriglyceridemia, lower levels of both total high density lipoprotein (HDL) and its subfraction HDL_2_, but also higher levels of HDL_3_ and small low-density lipoprotein particles. This review discusses the impact of diabetes and menopause to the lipid profile, challenges in lipoprotein subfractions determination and their potential contribution to the cardiovascular risk assessment in women. It is still unclear whether lipoprotein subfraction changes are a major driver of cardiometabolic risk and which modifications are predominant. Prospective trials with larger samples, methodological standardizations and pharmacological approaches are needed to clarify the role of lipoprotein subfractions determination on cardiovascular risk prediction and intervention planning in postmenopausal women, with or without DM.

## Background

Cardiovascular disease (CVD), particularly coronary artery disease (CAD) [1], is a major cause of death in women, who develop it about 10 years later then men [2]. Traditional risk factors are present at a high frequency in individuals with CAD but are lacking in a not negligible proportion. Risk calculators usually underestimate the real CVD risk in women and their CAD episodes are frequently fatal [3–5].

Hypercholesterolemia is the major driven cause for CVD in both sexes [6, 7] and its treatment has been associated with significant reductions in morbidity and mortality [8–10]. Postmenopausal women tend to deteriorate lipid profile that becomes more atherogenic than their premenopausal counterpart [11, 12]. After menopause, total cholesterol (TC) and low-density lipoprotein cholesterol (LDL-c) usually increase, and these changes are accompanied by a decrease in high-density lipoprotein cholesterol (HDL-c) and an increase in triglycerides (TG) [13, 14]. In addition to these major lipid abnormalities, also modifications in size and density of these lipoprotein particles are expected to happen after the loss of ovarian hormonal production [15–18]. This partially explains the increased cardiovascular risk in postmenopausal women [2, 19], particularly among those with an earlier onset of menopause [20].

Hyperglycemia contributes to the elevation of cardiovascular risk of populations. Increasing prevalence rates of type 2 diabetes mellitus (DM) have been attributed to aging, modern lifestyle and obesity epidemic, which predisposes to several metabolic disturbances linked by the insulin resistance [21–23]. In men and women with DM a typical dyslipidemia was described, characterized by hypertriglyceridemia, low levels of HDL-c and increased proportion of small-dense LDL particles, known to be more prone to oxidation [24–26]. Elevated glucose levels have also been associated with dysfunctional lipoprotein subfractions, contributing to a more atherogenic lipid profile in both sexes [27, 28]. Despite sharing these lipid abnormalities with the male sex, the diabetic woman has a more aggressive form of CAD and is more susceptible to death from CVD, mainly coronary events [29, 30], suggesting that her lipid profile should be even more deleterious. These observations indicate the need of additional quantitative and/or qualitative laboratory procedures—such as determinations of lipoproteins subfractions—to clarify some sex-related differences.

To date, there is paucity of data describing lipoprotein subfractions in postmenopausal diabetic women [1, 31, 32]. It is unclear whether accurate analysis of subfractions of the several lipoproteins could be associated with improved identification of women at higher risk, before and after menopause, with or without DM. In addition, menopausal hormonal replacement therapy (HRT) may impose unique risk to women. We review and discuss the differences in cardiovascular risk and lipoprotein subfractions in pre- and postmenopausal women and in diabetic ones. Understanding sex-related differences in lipid metabolism, as well as the impact of menopause and DM in women, may contribute to improve cardiovascular risk assessment in women. The keywords *postmenopausal and menopause, lipoprotein, lipoprotein subclass and subfractions, type 2 diabetes, analysis, cardiovascular risk* were selected for search in PubMed database, from 1980 to 2017, in English and/or Portuguese language.

### Cardiovascular risk in women

CAD and stroke have been the leading causes of death in both sexes accounting for 25.1% of the total mortality [33]. Even in the younger women, high mortality rates following myocardial infarction (MI) have been reported [34]. In recent years, improvements in hospital treatment [4] have contributed to a 30% decrease in the number of women dying from cardiovascular events in USA [35] although these still cause more deaths than all other causes combined. Estimates of cardiovascular risk and clinical trials are commonly based on unbalanced samples and selection bias has limited gender comparisons of outcomes. Female sex is notably under-represented in clinical trials which frequently have a predominance of the men [36]. Also, there is evidence that women are undertreated and have cardiovascular risk factors less controlled compared to men [37], specially the diabetic population [38].

Apart from methodological concerns, atherogenesis per se could affect men and women distinctly. It is known that atherosclerosis involves inflammatory and thrombotic processes. In premenopausal women, smaller lipid cores, less calcium, and fewer thin-capped atheromas were described, and estrogen-related anti-inflammatory effects on atherosclerotic plaques seem to contribute to their stabilization [39]. The plaque in women is shown to have less inflammatory components than in men which can implicate in slower development of vulnerable plaques. Young women with acute coronary syndromes often present plaque erosion, while men and older women frequently show the classical pattern of ruptured plaque followed by thrombosis [39]. In carotid arteries, lower atheroma burden and more stable plaques were described in women. Despite the ability of estrogen to stabilize the atheroma, prothrombotic effects of this hormone were reported. The reasons for sex-related differences in the development and progression of atherosclerosis are not completely understood [39–42].

Several scores have been proposed for cardiovascular risk assessment and the Framingham risk score is one of the mostly used [43–46]. It has been recognized that the Framingham risk score underestimates risk in women since those with subclinical atherosclerosis are often classified as at low risk [47]. In an update of this score, it was proposed that women should be classified as ‘‘high risk’’, ‘‘at risk’’ and ‘‘at ideal cardiovascular health’’. High-risk was defined by clinical evidence of CAD, peripheral artery disease and abdominal aortic aneurysm, or the presence of coronary risk equivalents, such as chronic kidney disease and DM, together with a 10-year predicted cardiovascular risk of ≥10%. At-risk women are those with at least one major risk factor [cigarette smoking, hypertension, dyslipidemia, obesity, poor diet, physical inactivity, family history of premature CVD, metabolic syndrome, evidence of advanced subclinical atherosclerosis (coronary calcification, carotid plaque, or increased carotid intima-media thickness), poor exercise capacity on treadmill test and/or abnormal heart rate recovery after stopping exercise, systemic autoimmune collagen-vascular disease (lupus or rheumatoid arthritis), history of preeclampsia, gestational diabetes, or pregnancy-induced hypertension]. “Ideal cardiovascular health” was defined by adequate total cholesterol and blood pressure levels, fasting plasma glucose and body mass index, with heart-healthy behaviours including healthy diet, smoking abstinence and regular physical activity [47, 48].

CVD incidence in premenopausal women is significantly lower than men at the same age (1 woman: 3–10 men), but increases to an extent that the rate becomes similar at the age 65 years and higher by the age 75 years [49]. Among the epidemiological studies that examined cardiovascular risk in women, the Nurses’ Health Study included one of the biggest sample [50]. This reported that 82% of coronary events could be attributed to the absence of a low-risk lifestyle. The INTERHEART study [6] revealed that nine risk factors accounted for 94% of the population attributable risk, including smoking, dyslipidemia, hypertension, DM, abdominal obesity, physical inactivity, low daily fruit and vegetable consumption, alcohol overconsumption, and a low psychosocial index (depression, locus of control, perceived stress, and life events). These are shown to be important risk factors for the development of CVD in both sexes.

In clinical settings, health care professionals commonly underestimate cardiovascular risk in women who are not as properly treated for CVD as men [47, 51]. Comparing sexes after MI, in every age, women are more likely to have a history of hypertension; however, concerning other risk factors, sex differences exist only before the age of 55, when women were more likely to have medical insurance, history of DM, heart failure or stroke, and higher Killip class on hospital admission [4]. Clinical symptoms of CAD also differ between sexes; men express classical symptoms such as angina, with pressure or squeezing to the chest, which can extend to the arms. Meanwhile, women tend to feel sharp, burning chest pain that can extend to neck, jaw, throat, abdomen or back and more frequently have atypical symptoms [52].

Sex differences could be raised concerning the efficacy of lipid lowering treatment. Statins have long been associated with reductions in total cholesterol, LDL-c as well as some increase in HDL-c concentration. Several meta-analyses reported significant reductions in cardiovascular outcomes with statins use for each 1 mmol/L decrease in plasma LDL-c [8, 9]. Accumulated evidence has consistently shown that statins are equally effective in both sexes in the control of dyslipidemia and reduction of cardiovascular morbidity and mortality [53, 54].

The deleterious impact of DM in cardiovascular morbidity and mortality is greater in women compared to men. In 2011, DM was responsible for 281,000 deaths in men and 317,000 in women, the majority from cardiovascular causes [55]. Despite being a strong risk factor for both sexes, a greater impact in mortality from CAD is seen in women than in men [56]. Its presence almost eliminated the sex-related difference in cardiovascular morbidity and mortality, approximating the risk level of the diabetic woman to the non-diabetic men [57]. Therefore, the diabetic woman needs special attention and optimized treatment of comorbidities to control risk factors and to decrease excessive cardiovascular mortality.

CVD is a major issue for women’s health most predominantly at older age, although the younger women have a higher chance of fatality following coronary events. Despite lower absolute incidence compared to men, high mortality rates indicate the need to improve risk prediction, early diagnosis and adequate treatment of risk factors and comorbidities to enhance women quality of life and survival. The increased mortality rates conferred by presence of DM are more prominent in the female sex. A careful analysis of these disparities between sexes is necessary.

### Lipoprotein subfractions: determinations and potentialities

Routinely, lipoproteins have been determined according to their molecular density (VLDL, LDL, and HDL) to assess cardiovascular risk. They have been classified by their size, charge, function, lipid core and apolipoprotein composition, and the resulting subgroups are called lipoprotein subfractions [58, 59].

A considerable proportion of individuals that suffer from cardiovascular events shows either few or none of the traditional risk factors [58, 60]. The assessment of lipoprotein subfractions and apolipoproteins (apo) represents a way to improve the cardiovascular risk prediction; in addition, they may enhance the accuracy of atherosclerosis detection, assist in treatment selection, and be useful for counselling first-degree relatives of patients with atherosclerosis [61].

Numerous methods for lipoprotein subfractions determination have been described, mostly for research purposes [61], such as analytic ultracentrifugation, vertical auto profile-II (VAP-II), density gradient ultracentrifugation, gradient gel electrophoresis, nuclear magnetic resonance (NMR) spectroscopy, immunoaffinity chromatography, 2-dimensional gel electrophoresis and ion-mobility analysis (Table 1). Heterogeneous techniques and nomenclature of lipoprotein subfractions limit data interpretation and study comparisons [59].

**Table 1:** Summary of main advantages and disadvantages of methods for lipoprotein subfractions determination

Method	Advantages	Disadvantages
Analytic ultracentrifugation	Precision and reproducibility	Unfeasible for clinical practice, due to low availability high cost and time consuming
Vertical auto profile-II	Simple procedures and high sensitivity	Low correlation to NMR and electrophoresis
Gradient gel electrophoresis	Determination of LDL and HDL size distribution directly from blood samples	Accuracy depends on correct standards and quality control Provides only the size of predominant species or average size
Linear polyacrylamide gel	Useful for clinical labs since it is simple and fast	High cost
Nuclear magnetic resonance spectroscopy	No need of physically separation of the subfractions and fast procedure	Dependent of mathematical assumptions
Immunoaffinity chromatography/ion mobility	Ability to isolate two HDL subfractions	Low availability and scarce data regarding efficiency

References: [58–77]

Analytic ultracentrifugation has been considered the gold standard of lipoprotein subclass analyses due to its precision and reproducibility, and used for validation of other techniques, but it is unfeasible for clinical practice [61]. This method is based on the lipoprotein ability to float when exposed to high gravitational forces. According to flotation rates, four LDL subfractions are grouped whose densities range from 1.025 to 1.060 g/mL [62].

The VAP-II uses a non-segmented continuous flow analyser for the enzymatic analysis of cholesterol in lipoprotein classes, allowing a profile analysis with only 40 µL of plasma [63, 64]. Five subclasses for HDL, four for Lp(a), four for LDL, two for IDL and three for VLDL can be identified. The absorbance curve provides the density distribution of lipoprotein classes and subclasses in the centrifuge tube [65]. The procedures are simple and sensitivity for the lipoprotein density classification is high. However, some studies have shown low correlation of VAP with NMR and electrophoresis [66].

The gradient gel electrophoresis determines LDL and HDL size distribution directly from blood samples. According to major peaks size and percent distribution, seven LDL subclasses, from larger buoyant LDL_1_, LDL_2a_ and LDL_2b_ to the smaller and less dense LDL_3a_, LDL_3b_, LDL_4a_ and LDL_4b_ can be detected [61]. Also, five HDL subclasses, ranging from small dense HDL_3c_, HDL_3b_, and HDL_3a_ to larger HDL_2a_ and HDL_2b_, can be determined. This method does not provide concentrations but the size of predominant species or average size [67]. The two-dimensional gel electrophoresis improved the ability of the gradient gel electrophoresis in recognizing new HDL subfractions: α1, α2, and α3, with sizes of 11.2, 9.51, and 7.12 nm, respectively [68]. Its use has been limited to specialized labs [61].

Lipoproteins subfractions determination can also be based on size and charge using linear polyacrylamide gel. The technique is simple and fast but expensive [69, 70].

NMR spectroscopy allows quantification of lipoprotein subfractions given that each lipoprotein particle in plasma has its own characteristic lipid methyl signal. NMR uses a library of lipoprotein spectra reference in a linear least-square fitting computer program [71]. From the shape of the composite plasma methyl signal, the program computes the subclass signal amplitudes. Particle sizes derive from the sum of the diameter of each subclass multiplied by its relative mass percentage [59, 61]. There is no need to physically separate the subfractions, which is a major advantage of the method. Lipoprotein subfractions identified are [71]:

- for VLDL: large VLDL/chylomicrons, medium VLDL, small VLDL
- for LDL, IDL, large LDL, medium small LDL, very small LDL
- for HDL, large HDL, medium HDL, small HDL

Immunoaffinity chromatography and the ion-mobility have been used for research purposes. The former is able to isolate two HDL subfractions through their content of apolipoprotein A-I and apolipoprotein A-II [61], while the latter determines concentrations of lipoprotein subfractions based on gas-phase differential electric mobility [59, 72].

The availability of several techniques and different parameters to express lipoprotein subfractions (concentrations, percent distribution of the HDL subclasses relative to the total or by average particle diameter) should explain part of the contrasting results on their association with CVD. The most consistent finding is the association of gradient gel electrophoresis-determined HDL subfractions [73]. The amount of large HDL identified by NMR has been correlated with the gradient gel electrophoresis HDL_2b_ results, but other NMR HDL components have shown weaker correlations [73].

Regarding LDL phenotype, substantial agreements among gradient gel electrophoresis, VAP, NMR, and ion-mobility have been described [74]. Using any of these four methods, association of small, dense LDL with coronary atherosclerosis progression was demonstrated [75]. Furthermore, gradient gel electrophoresis, NMR and ion-mobility confirmed that the associations were independent of standard lipid measurements. A recent study on the comparison of ultracentrifugation, a novel electrophoretic method and two independent methods of NMR indicated ultracentrifugation as the most precise method for LDL particle determination with the lowest coefficient of variation. The electrophoresis showed a close precision, whereas NMR showed the highest coefficient of variation [76].

Meanwhile, lipoproteins are heterogeneous even within each subclass and differ not only in size, charge and density, but also in their lipid and protein composition. Lipidomics and proteomics use mass spectrometry to identify and quantify lipid and protein content in a cell, tissue or organ, respectively [77–79]. These methods involve the use of complex technology in several research settings and may even help determine typical and abnormal lipoprotein composition [80, 81]. Changes in key components of lipoproteins under unusual circumstances, such as chronic inflammation and subclinical atherosclerosis, cause their remodelling, affect their functionality and contribute to the atherosclerotic process [82–84].

Evidence that certain lipoprotein subfractions enhance atherogenesis and increase cardiovascular risk emphasizes the importance of their determinations to improve the identification of those at higher risk [85, 86]. Determination methods differ by their basic principles, technology, complexity and accuracy. Such diversity limits to compare results and to assure the real contribution for the improvement in cardiovascular risk prediction.

Also, apolipoprotein determination has shown to improve cardiovascular risk assessment. Apo B100 concentration reflects the atherogenic lipoproteins (VLDL, IDL and LDL), while apo A-I has been considered a HDL surrogate. Apo B-to-apo A-I ratio provides a balance between the atherogenic and anti-atherogenic cholesterol particles and its usefulness as a predictor of cardiovascular events was demonstrated [87–89]. Lower apo B-to-apo A-I ratio was reported in premenopausal compared to postmenopausal women and men [90]. Lipoprotein (a) has a similar structure to LDL, containing one apo-B molecule combined with an apo (a), known to diminish plasminogen activation and fibrin degradation, favouring thrombosis. It has been considered an independent cardiovascular risk factor [91, 92]. There is no gender-related differences in lipoprotein (a) concentration, and a predictive value was observed only in men [93].

Standardization and cost reduction will be necessary for lipoprotein subfractions and apolipoprotein determinations reaching the clinical practice.

### Lipid changes following menopause and hormonal replacement therapy

Women experience modifications on lipid profile and metabolism from child to adult life, during pregnancies and following menopause. Aging itself is associated with an increase in LDL-c, in part due to a reduction in its catabolism by the liver. However, the higher levels of total cholesterol, LDL-c and apo-B found after menopause compared to premenopausal ones are not completely explained by aging [94]. A cross-sectional analysis of the Framingham Offspring Study [14], including 1597 women and 1533 men, showed higher LDL-c concentration in male sex, as expected. Additionally, in the postmenopausal compared to premenopausal women, increased LDL-c concentration was maintained after adjustments for age and several confounders.

Smaller denser Apo-B rich LDL particles are more frequent in postmenopausal women, while larger and buoyant LDL are decreased [16]. It is estimated that 14–30% of postmenopausal women have predominance of small dense LDL particles in contrast to only 5–7% in premenopausal counterpart [16, 95]. Lower HDL-c/total cholesterol and apo-AI/apo-B ratios [16, 95], as well as direct association of small LDL-c particles with TG levels, and inverse associations of HDL-c and Apo-AI with Apo-B were reported following menopause [95]. Increased TG rich lipoproteins are associated with higher proportions of small dense LDL. In postmenopausal period, affinity to the hepatic LDL receptor is reduced in small dense LDL-c that is more susceptible to oxidation, transendothelial transport and deposition in artery wall. This LDL subfraction has long been considered by the scientific community as an independent risk factor for CVD, although this is still controversial as some studies have failed to determine this association after several adjustments for confounding factors [58, 96–104]. Small dense LDL is also considered an independent risk factor for the development of type 2 DM [105], particularly in women [106]. Meanwhile, large HDL particles—also named HDL_2_—play an essential role on reverse cholesterol transport and are considered cardioprotective [66, 85, 107]. In postmenopausal women, the latter seemed to be diminished, with a predominance of cholesterol-depleted smaller HDL particles [18, 108–112]. These are not able to adequately transport cholesterol esters back to the liver, contributing to increased cholesterol concentrations in the blood.

In men, low levels of HDL_2_ particles (larger buoyant particles) have been associated with CAD indicating worse and diffuse lesions [113]. A cross-sectional analysis of more than 1000 women in UK showed that postmenopausal ones tended to decrease their total HDL-c concentrations together with a decrease in HDL_2_, without any difference in the HDL_3_ concentrations when compared to the premenopausal women [18]. Similar reductions in HDL_2_ were reported in high-risk postmenopausal women with untreated breast cancer [114]. Other studies have confirmed lower levels of large HDL_2_ particles following menopause suggesting that HDL_2_ concentrations might be influenced by the drop in female hormonal levels.

The role of sex hormones on lipid metabolism is supported by the demonstration that estrogenic therapy prevents decrease in LDL-c and increases in TG and VLDL-c concentration after menopause. Mechanisms by which female hormones interfere on lipid metabolism have been largely investigated. Estrogen is shown to increase both LDL receptor population in the liver, together with hepatic production of TG rich lipoproteins. Some authors have proposed that the lack of estrogen after menopause contributes to hypertriglyceridemia, low HDL-c and a predominance of small dense LDL particles, like the abnormalities seen in the metabolic syndrome [115]. This lipid profile is found in 15–25% of postmenopausal women and might in part be responsible for their increased cardiovascular risk [115]. The very large lipid database (VLDL 10B) study [116], in which more than a million-people had their lipoprotein subfractions measured by density gradient ultracentrifugation, supported that, after middle age, women presented a shift towards a more atherogenic lipid profile.

These findings have raised questions about the utility of hormonal replacement therapy (HRT) to prevent lipid metabolism abnormalities following menopause which could help in the prevention of CVD. Several clinical trials were conducted to investigate the effects of different schemes of HRT on the lipid profile after menopause [117–120], but those using accurate methods for the determination of subfractions of lipoproteins are less numerous [121, 122]. In one study, 38 postmenopausal Brazilian women with formal indication for HRT were treated with continuous doses of 0.625 mg of conjugated equine estrogen (CEE) with (if they had uterus) or without 2.5 mg of medroxyprogesterone for 12 weeks. Lipoprotein subfractions were measured using an NMR spectroscopy at baseline and after treatment. Significant increases in larger VLDL and HDL particles, together with a decrease in the smaller HDL and VLDL particles were observed, but treatment did not induce significant differences in LDL subfractions [123].

Another trial evaluated the effect of estrogen alone or combined with medroxyprogesterone (1 mg of 17β-estradiol and/or 0.625 mg of CEE) for 3 months in 43 postmenopausal women [124]. Combined therapy resulted in a significant increase in the proportion of bigger HDL particles in circulation, also diminishing the absolute amount of smaller HDL particles. Other trials with estrogen alone in surgically induced menopause have shown a tendency for an increase in HDL and HDL_2_, but a variety of results were found for LDL particles [118–121]. Different HRT regimens, such as natural vs synthetic, transdermal vs oral, cyclic vs continuous, different progestogens or estrogens and doses have also been tested, but modifications in both lipid and lipoprotein subclasses are inconsistent across trials.

An interesting analysis of 243 postmenopausal women from the Healthy Women Study confirmed higher levels of large HDL particles measured by NMR spectroscopy between HRT users as compared to nonusers [125]. Despite lower levels of LDL-c, there were no differences in LDL subclasses or in coronary artery calcification (CAC) between the groups. As expected, having detectable CAC was associated with worse traditional lipid profile and increased atherogenic subfractions. Although an HRT-dependent shift on the proportions of lipoprotein subfractions could be expected in postmenopausal women, trials have not shown any benefit in cardiovascular morbidity or mortality [126–128]. Only in a subset of younger women who initiated on HRT immediately after menopause some beneficial effects were detected [129]. Scientific societies have not recommended estrogen replacement aiming at treating dyslipidemia or reducing cardiovascular risk in postmenopausal women [130–132].

Since aging and menopause provoke lipid changes (decreased HDL, especially HDL_2_, increased small dense LDL and TG) that elevate cardiovascular risk in women partially controlled by HRT, several open questions need to be addressed to improve the prognosis of the atherosclerotic disease.

### Disturbances in lipid profile and lipoprotein subfractions in diabetes and in postmenopausal diabetic women

Type 2 DM commonly coexists with obesity and both are characterized by states of low-grade inflammation and insulin resistance. Type 1 macrophages accumulated in the hypertrophic adipose tissue potentiate the pro-inflammatory cytokines secretion. Efflux of free fatty acids into circulation and the hepatic insulin resistance are responsible for the dyslipidemia in this condition [133, 134]. Molecular mechanisms of the lipid metabolism disturbances in DM involve microRNAs, that are non-coding RNA molecules which regulate gene expression post-transcriptionally [135]. When microRNAs bind to their complementary sites at the 3′-untranslated regions of the target messenger RNAs (mRNAs) results in mRNA translational and repression or transcript degradation [136, 137]. They have been proven to play important role on insulin resistance and on the regulation of liver metabolism affecting circulating lipids (miR-122, miR-33a, miR-33b) and lipoprotein receptor. The relationship between insulin resistance and hypertriglyceridemia has been recognized, whereas through microRNA miR-34a, hypertriglyceridemia seems to favor the onset of DM [138, 139].

Obesity and impairment in glucose tolerance are frequent pathophysiological conditions that generate lipid-related cardiovascular risk in women following menopause. As chronic inflammatory states, these conditions contribute to lipoprotein remodelling, compromising its function. Meanwhile, reduced estrogen levels contribute to a decrease in insulin sensitivity and aggravate metabolic disturbances [140]. Therefore, postmenopausal obese type 2 diabetic individuals are prone to a combination of disorders that markedly increases the risk of dying from cardiovascular events [141, 142]. Obesity-induced efflux of free fatty acids provokes insulin-mediated skeletal uptake of free fatty acids and increased liver exposure, which results in a rise in hepatic secretion of VLDL, together with a retarded clearance of VLDL and chylomicrons, contributing to hypertriglyceridemia. This pattern of large VLDL, named VLDL_1_, results in increased precursors of small dense LDL-c [143].

The typical pattern of dyslipidemia in DM—characterized by hypertriglyceridemia, low HDL-c and high small dense LDL-c levels—does not differ between sexes [144]. The HDL-c catabolism that occurs by the hepatic lipase and TG enrichment is elevated in conditions of insulin resistance [145]. Consequently, there is a reduction in HDL-c—that is predominantly from the HDL_2b_ subclass—as well as a relative or absolute increase in the smaller denser HDL_3b_ and HDL_3c_ [143]. Elevated non-HDL-c and predominance of small dense LDL particles to large buoyant LDL, known as phenotype B [143, 146], raise atherogenicity even in near-normal limits of LDL-c. As these particles are prone to oxidative modification, oxidized LDL is more frequently found in diabetic individuals, contributing to accelerate atherogenesis.

Small dense LDL particles have reduced affinity to LDL receptors and a prolonged plasma residence time, which could result in an increment in LDL_3a_ and LDL_3b_ and a decrement in LDL_1_ and LDL_2a_ [143]. Of note, the opposite and desirable profile, with higher concentration of large buoyant LDL, has been called phenotype A [143, 146]. TG enrichment of these particles (VLDL and LDL) is due to the action of cholesteryl ester transfer protein (CETP), and hepatic lipase hydrolysis of TG and phospholipids [143, 147].

In addition, abnormalities on scavenger receptor class BI (SR-BI), that promotes selective uptake of HDL cholesteryl esters (HDL-CEs) into cells, have been described in the type 2 DM. An overexpression of SR-BI in the liver accompanied by a reduction of HDL-c levels were reported [148]. In contrast, genetic deletion of SR-BI resulted in increased HDL-c and atherosclerosis. These HDL-c molecules seemed to have an altered composition, including a shift toward large, buoyant HDL particles, and a significant increase in plasma apo A-I, but not apo A-II in HDL particle [149].

Consequences of insulin resistance can be present in individuals with the metabolic syndrome even before the clinical diagnosis of DM [143, 145]. Hyperglycaemia and hypoadiponectinemia are involved in the pathophysiology of the diabetic dyslipidemia, but several questions remain unanswered [145].

Incidence of type 2 DM elevates after menopause [150] and that postmenopausal diabetic women are at increased cardiovascular risk compared to nondiabetic women at the same age and hormonal status [30]. Such risk is strongly related to modifications in the lipid metabolism which are dependent of both, menopause per se as well as the diabetic condition. For our best knowledge, the deleterious impact on lipid metabolism due to the presence of DM is similar in men and postmenopausal women.

**Fig. 1:**
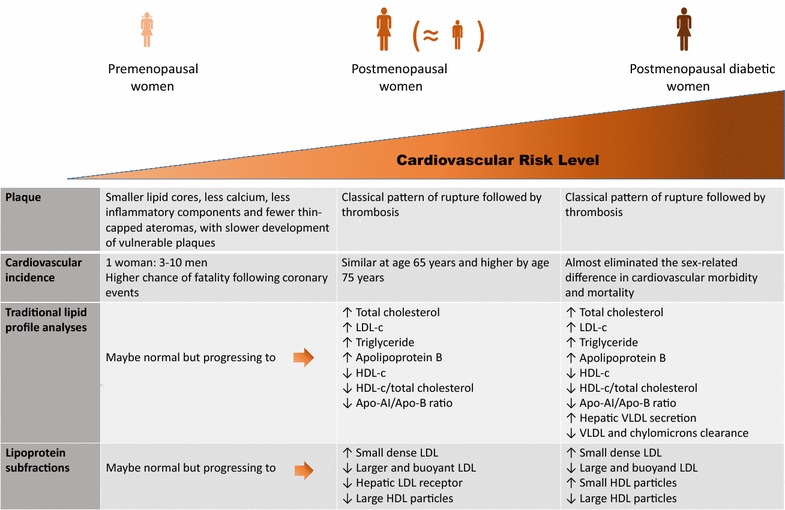
Main characteristics of structural and functional abnormalities of lipid metabolism during atherogenic process and aging and the impact of diabetes mellitus

The increased risk for atherosclerosis in postmenopausal diabetic women depends on low HDL-c levels, hypertriglyceridemia and predominance of small dense LDL particles [151]. Additionally, type 2 DM clusters with other disturbances from the spectrum of the metabolic syndrome, contributing to an elevated cardiovascular mortality [152]. Interestingly, the deleterious impact of DM in the LDL particle size seems to be greater in the diabetic women than in men [153, 154] and postmenopausal diabetic women exhibited decreased large HDL particles (HDL_2_) levels together with increased small HDL particles compared to normoglycemic women after menopause [31]. Figure 1 summarizes the main characteristics of structural and functional abnormalities of lipid metabolism during atherogenic process and aging and the impact of diabetes mellitus.

Meanwhile, the hypothesis that estrogen therapy could alter lipids and improve cardiovascular risk profile and outcomes has been tested in both, diabetic and non-diabetic women [123, 155–168]. Despite many studies that investigated the HRT effects on cardiovascular risk factors in postmenopausal diabetic women, just a few evaluated lipoprotein subfractions with conflicting results. Some authors described a significant increase in total HDL, predominantly on the HDL_2_ subfraction, after intervention with combined HRT [168], while others failed to demonstrate any impact on HDL or LDL subfractions [32, 166]. Due to the limited sample size and different HRT schemes used, studies available only generated hypothesis.

The effect of HRT on glucose homeostasis remains questionable [158]. A systematic review which included 16 trials with 17,971 postmenopausal women with type 2 DM demonstrated that estrogen replacement diminishes DM incidence and improves glycemic control [169], but there is no consensus yet.

To summarize, limited data on lipoprotein subfractions distribution in postmenopausal diabetic women, with or without dyslipidemia, are available. Different pharmacological approaches to ovarian failure still deserve comparisons, as well as different analytical methods to measure lipoprotein subfractions. Glycemic control level may add a confounding factor among comparisons contributing partially for inconsistent results.

### Worth of measurement of lipoprotein subfractions to the cardiovascular risk assessment in women

To date, there is insufficient evidence to recommend lipoprotein subfractions determination in clinical practice in both sexes at lower or higher cardiovascular risk [169]. Evidence that this measurement would impact on lipid-lowering treatment strategies is lacking either [170].

A small prospective nested case–control study in normal middle-aged women has previously demonstrated that baseline particle concentration was more predictive of future cardiovascular events than LDL particle size [171]. On the other hand, an analysis of 286 postmenopausal women from the Healthy Women Study confirmed an independent association of small dense LDL with higher CAC scores, suggesting a benefit from the addition of lipoprotein subfraction measurement for CVD prediction in this subset of individuals [172].

The largest prospective trial available included 27,673 healthy women followed for 11 years [173]. Traditional lipid profile and NMR-determined lipoprotein subclass number and size were measured at baseline. No extra benefit on cardiovascular risk prediction with lipoprotein subfractions measurement after adjustment for non-lipid risk factors was obtained [173]. Finally, a recent systematic review of 24 studies, in which the impact of LDL particles for cardiovascular outcomes was examined in both sexes, reported similar findings [174].

In summary, controversies in this matter persist [175] and it is questionable whether determination of lipoprotein subfractions could be useful in clinical settings. Several techniques for measurement are available, costs of the assays are high and the incremental benefit beyond traditional lipid measures may be minimal. Prospective studies demonstrating that advantages of lipoprotein subfractions to traditional lipid profile in the context of primary and secondary prevention of cardiovascular outcomes are needed.

## Final remarks

Despite the lower incidence of CVD in adult women compared to men, their sex-related protective effect vanishes after menopause. This phase of women life per se imposes deterioration of their lipid profile and weight gain is a frequent manifestation that could aggravate their predisposition to metabolic disturbances. The cardiovascular risk scores and health care professionals commonly underestimate their risk, and higher mortality and morbidity after coronary events have been reported in women. Consequently, women are less properly treated for CVD than men.

The deleterious impact of type 2 DM in cardiovascular risk may be superior in women compared to men, emphasizing the importance of improving the risk assessment, especially in postmenopausal diabetic women.

Since plasma lipoproteins constitute a major cardiovascular risk factor, a deeper analysis of their subfractions might contribute to understanding why lipid-dependent cardiovascular risk in women is increased. A more atherogenic lipid profile—hypertriglyceridemia, lower levels of both HDL-c and HDL_2_, higher levels of both HDL_3_ and small dense LDL—are usual after menopause, and modifications in lipoprotein subfractions are also expected in the presence of hyperglycemia. Therefore, postmenopausal diabetic women should be aggressively treated against dyslipidemia as well as against other risk factors.

Nowadays, no evidence supports that replacement of ovarian hormones has benefits in reducing cardiovascular events and mortality in different subgroups of women.

Finally, prospective trials including large samples of postmenopausal women, with or without DM, at different treatments and metabolic control, should be conducted to clarify whether lipoprotein subclass analysis would improve identification of higher-risk individuals. Considering that these determinations are expensive, cost-effectiveness studies are also necessary to address the worth of the addition of lipoprotein subfraction analysis in clinical practice.

## Abbreviations

CVD: cardiovascular disease
CAD: coronary artery disease
TC: total cholesterol
HDL-c: high density lipoprotein cholesterol
LDL-c: low density lipoprotein cholesterol
TG: triglycerides
DM: diabetes mellitus
HRT: hormone replacement therapy
MI: myocardial infarction
VAP: vertical auto profile method
NMR: nuclear magnetic resonance
Apo: apolipoprotein
CEE: conjugated equine estrogen
CETP: cholesteryl ester transfer protein
